# A new 3D finite element-based approach for computing cell surface tractions assuming nonlinear conditions

**DOI:** 10.1371/journal.pone.0249018

**Published:** 2021-04-14

**Authors:** Silvia Hervas-Raluy, Maria Jose Gomez-Benito, Carlos Borau-Zamora, Mar Cóndor, Jose Manuel Garcia-Aznar

**Affiliations:** 1 Department of Mechanical Engineering, University of Zaragoza, Zaragoza, Spain; 2 University Center for Defense, Zaragoza, Spain; 3 Biomechanics Section, Department of Mechanical Engineering, KU Leuven, Leuven, Belgium; University of New South Wales, AUSTRALIA

## Abstract

Advances in methods for determining the forces exerted by cells while they migrate are essential for attempting to understand important pathological processes, such as cancer or angiogenesis, among others. Precise data from three-dimensional conditions are both difficult to obtain and manipulate. For this purpose, it is critical to develop workflows in which the experiments are closely linked to the subsequent computational postprocessing. The work presented here starts from a traction force microscopy (TFM) experiment carried out on microfluidic chips, and this experiment is automatically joined to an inverse problem solver that allows us to extract the traction forces exerted by the cell from the displacements of fluorescent beads embedded in the extracellular matrix (ECM). Therefore, both the reconstruction of the cell geometry and the recovery of the ECM displacements are used to generate the inputs for the resolution of the inverse problem. The inverse problem is solved iteratively by using the finite element method under the hypothesis of finite deformations and nonlinear material formulation. Finally, after mathematical postprocessing is performed, the traction forces on the surface of the cell in the undeformed configuration are obtained. Therefore, in this work, we demonstrate the robustness of our computational-based methodology by testing it under different conditions in an extreme theoretical load problem and then by applying it to a real case based on experimental results. In summary, we have developed a new procedure that adds value to existing methodologies for solving inverse problems in 3D, mainly by allowing for large deformations and not being restricted to any particular material formulation. In addition, it automatically bridges the gap between experimental images and mechanical computations.

## Introduction

Many cellular processes, such as angiogenesis, wound healing and cancer metastasis, are driven by physical forces exerted by cells and are therefore important for the purpose of understanding and manipulating these biological processes [1–4]. Cells generate internal tensile forces through actomyosin interactions and exert tractions on the underlying extracellular matrix (ECM) [5–8]. Due to the key roles of such physical forces, it is essential to achieve a complete knowledge of cell traction force regulation as well as to be able to measure these forces [9, 10].

The current understanding of cell-matrix interactions is based primarily on *in vitro* studies of focal adhesions and other adhesive structures on planar two-dimensional (2D) tissue culture substrates [11]. Nevertheless, cell behaviours in three-dimensional (3D) environments have shown significant differences [12–14]. Indeed, the ECM is a critical component, providing biophysical and biochemical cues to resident cells via cell-matrix interactions [4]. Cells embedded in a 3D matrix exhibit dramatically different morphologies, cytoskeletal organizations and focal adhesion structures compared to those in 2D substrates. Moreover, when cells migrate in a 3D matrix, they must overcome not only the adhesion forces but also the resisting forces imposed by the surrounding matrix [15]. Resisting forces mainly arise from steric effects. This steric hindrance, in turn, depends on the matrix properties (e.g. pore size and fiber stiffness [16, 17]) as well as cell properties (e.g. cell size or cell stiffness [18–20]). Some studies, such as that of [21], have demonstrate that cells can compensate for the increased steric hindrance in a dense 3D network and maintain their invasive behaviour.

In recent decades, several methods to quantify cell traction forces have been developed, both in 2D and 3D environments. Forces are estimated from the measurements of other mechanical quantities, such as the properties of materials and the displacement field caused by cells while interacting with their substrate.

Examples of these methods are the cantilever and micropillar methods, the use of intracellular tension sensors and traction force microscopy (TFM) [22]. In the former method, surface forces are measured using calibrated cantilevers. These structures are incorporated into the system so that, as the tissue contracts, the cantilevers bend. The micropillar technique allows us to map the forces at the subcellular level. Nevertheless, micropillars exhibit a unique surface topology, which influences the cell adhesion structures and can affect the magnitudes and distribution of cellular forces [23, 24]. The second method, which relies on the use of intracellular tension sensors, has been developed in recent years, and it aims to report cytoskeletal stresses in cells. For intracellular sensors, one can use a Förster resonance energy transfer (FRET), which means that fluorescence decreases as the linker is stretched [25]. The latter method is the one we are dealing with. TFM can be implemented in 3D environments, and it allows for the measurement of tractions exerted by cells in their natural states.

Typically, confocal microscopy images are used to determine cell-induced displacements as well as the cell geometry [26]. The displacement field can also be recovered using second harmonic generation (SHG) microscopy techniques. Then, the displacement field is computed from a pair of images (or image stacks if 3D) by taking advantage of the fluorescent bead positions. The first image/stack usually corresponds to the stressed state of the cell-matrix environment. After that, an actin disruption drug is applied to the cell, inducing it to enter its relaxed state. The second set of images corresponds to this step [5]. After that, the displacement of the beads is determined by applying particle tracking techniques between both stacks of images [27].

To recover the cell traction forces, normally, the follow-up approach is to solve an inverse problem that reconstructs the best combination of forces that leads to the measured displacement field [5, 28]. However, this requires an appropriate constitutive model that defines the relationship between stress and strain [29]. An alternative to solving the inverse problem is the direct method that constructs the stress tensor by direct mapping from a strain tensor calculated from the image data [3, 30].

Problems in 2D environments are solved analytically using the Boussinesq solution in Fourier space [31, 32]. Nevertheless, the applicability of this approach is limited to thick 2D substrates under small strains. In recent years, the resolution of the computational TFM problem has been widely extended to 3D. To solve the 3D-TFM problem, Green’s function formalism can be used in the linear case. First, [33] developed an approach that can be applied to real cell geometries embedded in 3D gel matrices. They approximated Green’s function via finite elements (FEMs) to determine a solution, combining analytical techniques with computational methods. After that, a minimization problem was solved to determine the traction force field. Later, [34] considered a nonlinear constitutive model but did not account for nonlinearities in the strain and in the geometrical description of the problem. A few other attempts have been made to study the nonlinear properties of materials [35, 36]. Recently, [12] presented a technique for a 3D full-field quantification of large cell-generated displacements. They applied free form deformation-based image registration [37] to compute full-field displacements and strains. [38] presented an iterative methodology to solve inverse problems in TFM. This methodology relies on recursive optimization based on an objective function defined by least-squares minimization of the difference between the target and the current computed deformed configuration of the cell. 3D techniques have increased in sophistication and now feature high spatial resolution and advanced computational formalisms to connect displacement information to material constitutive laws [3, 38, 39].

**Fig 1:**
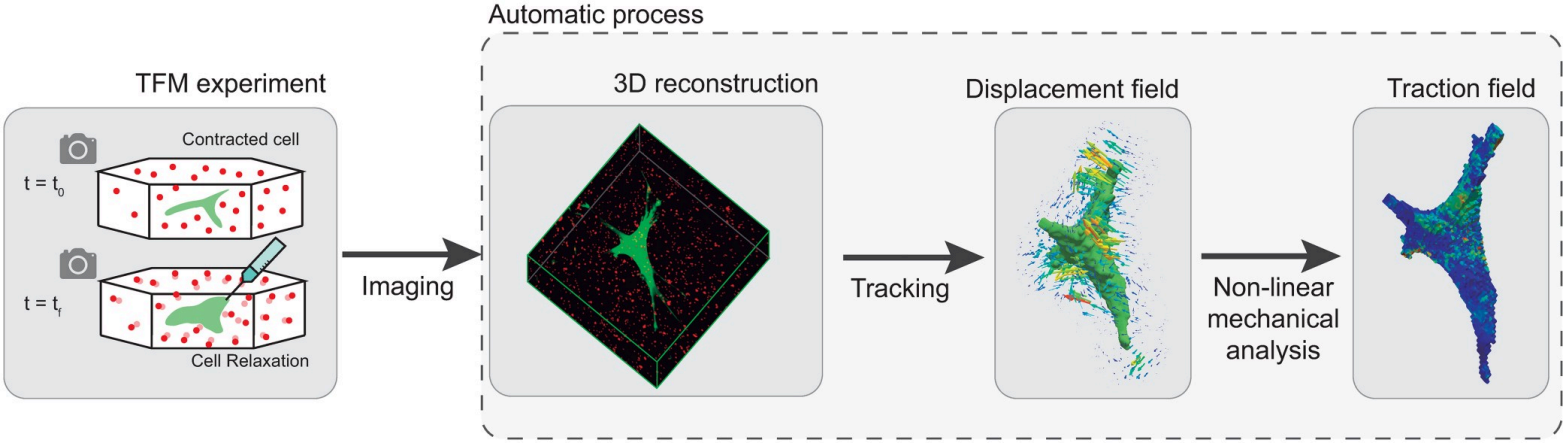
Workflow for 3D TFM and the developed numerical algorithm. Cellular tractions are obtained via a 3-step method. First, two pictures are captured during the experiment. Then, a 3D segmentation is obtained through the imaging process. The tracking method computes the displacement field and the cell geometry. Finally, the traction field is obtained via the resolution of the inverse problem.

In this study, we present a novel methodology for computing cellular surface tractions, including in finite strain scenarios (see Fig 1). For that purpose, a general FE-based methodology is proposed with the aim of estimating the traction forces exerted by a cell from the experimental bead displacement measurements. The cell geometry is reconstructed via the laser scanning of confocal microscopy images. The z-stacks obtained are automatically segmented to generate 3D models of both the cell and ECM geometries. These geometries are automatically meshed and used to generate the initial FE model used in the subsequent mechanical simulations. ECM displacements are computed from the experiments and extrapolated to the FE mesh. This displacement field is inferred from the measurement of the 3D position changes of fluorescent beads scattered throughout the ECM between two different time points. To perform the simulations, both geometric and material nonlinearities must be considered. We propose two different cases: the first is a direct theoretical case (both the displacement and force fields are known), and it is used to validate the FE methodology. In the second case, we apply the methodology to a real experiment to infer cell surface tractions from measured displacements. The procedure presented here aims to improve several shortcomings of existing methodologies with regard to solving inverse problems. Namely, this work automatically recovers the displacement field directly from the 3D TFM experiments and uses it as an input for the inverse problem solver, thereby bridging the gap between the experimental data and the computational results. Moreover, traction forces are recovered under the hypothesis of large deformations, and the methodology is not restricted to linear materials. In fact, our method is applicable to any kind of geometry and material property set.

The present work is organised as follows. First, the methodology is presented, ranging from experimental trials to the numerical algorithm developed. Two different analyses are performed to first validate the methodology and then to apply it to a real TFM experiment. We show that the proposed methodology successfully computes the surface tractions introduced in the simulated problem and those measured experimentally.

## Materials and methods

### Cell culture and preparation of 3D collagen gels

Normal human dermal fibroblasts (NHDFs, obtained from Lonza) transfected with lentiviral particles and isolated by cell sorting were kept in 25 *cm*^2^ cell culture flasks with Fibroblast Growth Medium-2 (FGM-2 BulletKit, Lonza) under cell culture conditions (37°C, 5% CO_2_ and 95% humidity). GFP-expressing fibroblasts (NHDF-GFP) were passaged every second day using 0.05% trypsin/EDTA. To prepare 200 *μl* of 2 mg/ml collagen type I hydrogels, we carefully mixed 133.3 *μl* of rat tail collagen (3 mg/ml, BD Bioscience), 20 *μl* of 10x Dulbecco’s phosphate buffered saline (DPBS, Gibco), 8 *μl* of red carboxylate-modified fluorescent latex beads with 1 *μm* diameters (FluoSpheres^®^, Invitrogen, F8821), 2.3 *μl* of distilled water and 6.4 *μl* of sodium hydroxide (0.5 N NaOH, Sigma) to adjust the pH of the collagen solution to 7.4. 30 *μl* of NHDF-GFP cells suspended in a complete cell culture medium (FGM-2, Lonza) were carefully mixed with the final collagen solution before gel polymerization was performed at a ratio of 50000 cells/ml. All ingredients were kept on ice during the collagen preparation process. 20 *μl* of the final cell-collagen solution was pipetted through a microfluidic device and polymerized in a tissue culture incubator at 37°C, 95% relative humidity and 5% *CO*_2_ for 20 minutes. After polymerization, 300 *μl* of the complete cell culture medium was added to both lateral channels to prevent the collagen gel from becoming dehydrated. For more information about the critical parameters and troubleshooting for the collagen gel preparation process, see [27].

### Live cell imaging inside a microfluidic device

After overnight cell-collagen solution coincubation within microfluidic devices, individual live cell imaging was performed. Confocal microscopy imaging (Nikon C1 confocal microscope) with a Plan Fluor 40x/1.30 Oil objective was used to obtain a set of planar images that was later used to reconstruct the 3D individual cell geometries. The image stack was recorded by exciting the samples with a 488 nm argon laser (green channel) and a 561 nm He-Ne laser (red channel), with a distance from the confocal z-sections of 0.63 *μm*.

With a typical number of z-stacks (130) and 512x512 pixels in the x-y plane, the voxel dimensions selected for the cell image acquisition were 0.63 *μm* horizontally in the x and y directions and 0.63 *μm* vertically in the z direction. The selected cell was always located at least 50 *μm* away from the top or bottom of the gel surface or from neighbouring cells to avoid contact with the microfluidic device or cell-cell attachments. Before selecting a cell for imaging purposes, it was also checked that the hydrogel in the region of interest did not show any irregularities. To ensure the stability of the physiological imaging conditions within the imaging chamber, cell culture conditions (37°C, 5% *CO*_2_ and 95% humidity) were maintained during all time lapse recordings.

### 3D traction force microscopy assay

To compute the force-induced deformations of the biopolymer network, the fluorescent beads were imaged by means of confocal microscopy before and after the cell traction forces were relaxed with cytochalasin-D, as previously described in [27]. One image stack was recorded before the cell traction forces were relaxed, and a second image stack was recorded 30 min after the addition of cytochalasin-D. The first image stack represents the deformed state, whereas the second image stack represents the undeformed force-free configuration of the matrix. Before the addition of cytochalasin-D, the cell culture medium was removed by manual aspiration from both lateral channels of the microfluidic device. Subsequently, 120 *μl* of 40 *μM* cytochalasin-D was added into the condition channel to induce the diffusion of the substance through the central channel of the device, where the cells were located.

### Matrix displacement quantification and cell geometry definition

In the experimental setup, the matrix displacement field was inferred from the measurement of the 3D position changes of the fluorescent beads scattered throughout the gel between two different time points (namely, the contraction and relaxation phases). Images were taken throughout the depth of the gel (z-axis) every 0.63 *μm*. Following the methodology proposed by [40], the bead positions in the first image stack were initially identified via a local intensity maximum above a specific threshold. Then, a 7x7x7 pixel subvolume (0.5x0.5x0.5 *μm*) around each bead position was selected. To obtain the subpixel accuracy, the center of mass (in this case, the squared intensity) of the subvolume around each bead was iteratively computed until convergence was achieved. Finally, these subvolumes were shifted in the second image stack (by subpixel increments) until the squared differences of the pixel intensities were minimised. The cell geometry was obtained by automatic 3D threshold segmentation. Then, the obtained pixels were automatically interpolated to a 3D voxel mesh, where the experimentally obtained displacements were interpolated via a custom-written image processing algorithm in MATLAB.

To avoid artifacts at the image borders as well as the possible effects of neighbour cell forces, we only kept the displacements of the cell action area, which was assumed to be 10 *μm* around its surface, therefore zeroing the bead displacements that were measured beyond this frontier. Additionally, to prevent discontinuities between adjacent pixels, we applied a decreasing quadratic function to smooth the displacements at the edge of the cell action area.

### Determining cell surface tractions under large deformations

To calculate the surface tractions induced by a cell embedded in the ECM, a new approach is presented. Unlike in previous models, we work under a hypothesis of large deformations (*ε*> 0.1). To demonstrate the robustness of the model, we test it under an extreme case with strains above 110%.

First, a brief introduction of the basic kinematic quantities of geometrically nonlinear continuum mechanics is presented. Let Ω_0_ denote the material configuration or the undeformed shape of a continuum body parametrised by the material coordinates **X** at time *t* = 0 and let Ω_*t*_ denote the corresponding spatial configuration or deformed shape parametrised by the spatial coordinates **x** at time *t*.

In the direct mechanical formulation, the material configuration is known, and the objective is to determine the direct deformation map ***φ*** as **x** = ***φ***(**X**): Ω_0_ → Ω_*t*_. However, in the inverse mechanical formulation [41], the spatial configuration is given, and the inverse deformation map **Φ** must be determined as **X** = **Φ**(**x**): Ω_*t*_ → Ω_0_

We consider a cell embedded in the ECM. The cell geometry, reconstructed from confocal images, defines the cell surface, which in turn separates two domains: the cell itself and the surrounding matrix. The domain $\Omega \subset {I\;R}^{3}$ represents both the ECM in which the cell is embedded and the cell itself, with Ω_*cell*_ being the cell domain and Ω_*ECM*_ being the ECM domain.

The surface traction field (**T**_*real*_) in the undeformed configuration is calculated along the cell surface, and it is defined as the boundary Γ_*s*_ = Ω_*cell*_ ∩ Ω_*ECM*_.

**Fig 2:**
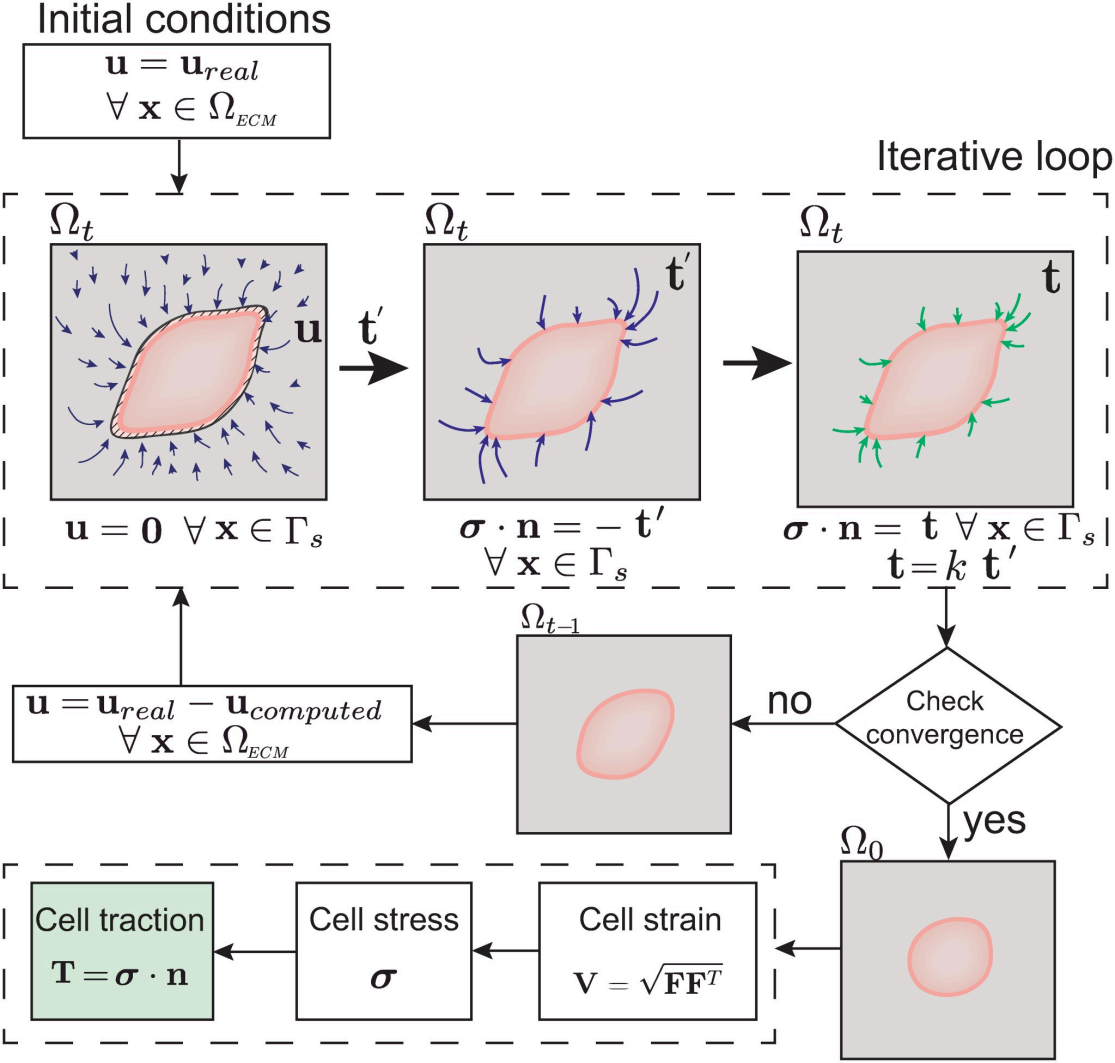
Scheme of the iterative workflow. During the iterative loop, three different analyses are run to solve the inverse problem. In the first analysis, the displacement field (**u**) is applied, and the surface (Γ_*s*_) is encastered. Thus, a set of intermediate reaction forces (**t**′) is obtained. The intermediate reaction force field obtained is applied in the opposite direction. This results in an estimated displacement field (**u**′). The ratio between **u** and **u**′ is used to adjust the previous traction force field to a new one (**t**), resulting in a new deformed configuration. This process is repeated until convergence is achieved. To extract the cell surface traction field, the logarithmic strains are postprocessed.

Let **u**_*real*_ be the known displacement field measured from the TFM experiments as the displacements of the fluorescent beads embedded in the ECM. Our goal is to solve the inverse problem estimating the traction forces **T**_*real*_ at the material configuration. The cell exerts these surface tractions on the extracellular matrix to generate the known displacement field **u**_*real*_. To this end, we have developed an iterative numerical method that is mainly composed of three consecutive mechanical analyses (see Fig 2).

First, the displacement field **u**_*real*_ is imposed on the matrix in the spatial configuration, constraining both the cell surface and cell body so that **u** = **u**_*real*_ ∀ **x** ∈ Ω_*ECM*_, and **u** = **0** ∀ **x** ∈ Γ_*s*_. Thus, the first traction force field for the current configuration (Ω_*t*_), **t**′, is estimated.

Second, the previously computed traction force field **t**′ is applied at the cell surface, and thus, an estimated displacement field **u**′ is obtained. The ratio between the sums of the real and estimated displacement magnitudes results in a coefficient *k*. Therefore,

$$\begin{array}{c} k=\frac{\sum_{i=0}^{N}\|u_{real}\|}{\sum_{i=0}^{N}\|u^{\prime }\|} \end{array}$$ (1)

where *N* is the total number of points in the discretization.

Subsequently, the traction forces are adjusted by minimizing the mismatches between the computed and real displacements. We then estimate a possible solution for the cell traction field as **t** = *k* ⋅ **t**′. Finally, in the third analysis, the new traction force field is applied, ***σ*** ⋅ **n** = −**t** ∀**x** ∈ Γ_*s*_, and we obtain a new displacement field **u**_*computed*_.

Therefore, the obtained deformed configuration is compared to the original (experimentally measured) deformed configuration. To do this, **u**_*real*_ is quantitatively compared to **u**_*computed*_ for every point in the discretized domain according to:

$$\begin{array}{c} err=\frac{{\left\|u_{measured}-u_{computed}\right\|}_{2}}{max({\left\|u_{measured}\right\|}_{2})} \end{array}$$ (2)

where the norms are the Euclidean norms.

If the error is less than a threshold *δ*, the algorithm has converged to a solution. If the error is still large, the obtained deformed configuration is updated and then imported to the start of the new loop (see Fig 2). When a new loop starts, a new set of displacements is applied, **u** = **u**_*real*_ − **u**_*computed*_, in the new configuration Ω_*i*_.

In this way, the geometry obtained steadily approaches the real undeformed geometry as the number of iterations increases. By updating the geometry in each deformation, we adopt an updated Lagrangian approximation, whose formulation is also applied in the finite element analyses, to fulfill the hypothesis of finite deformations.

The traction forces obtained during each iteration are stored and updated to the new configuration (Ω_*t*_), since the final resulting forces will be computed based on the historical traction forces and the final obtained forces.

Therefore, once the analysis is finished, a postprocessing code is executed to obtain the surface tractions in the final configuration (which is the real undeformed configuration) and the global error.

### Numerical implementation

#### Geometric definition

The 3D cell geometry is automatically segmented and discretised in a voxel mesh from the images taken during the TFM experimental assay. In addition, to simulate the cell embedded in the ECM, the latter volume is modeled as a rectangular prism. The cell is located in the middle of this volume. All the exterior surfaces of the rectangular prism are constrained (no displacements are allowed).

Before initializing the mechanical analysis, the voxel mesh is converted into a tetrahedral mesh via the Tetgen software [42] to facilitate automatic remeshing. Therefore, an automatic transformation is performed after discretizing the image-obtained geometry to obtain a tetrahedral mesh. The resulting mesh has a minimum volume per element of 2, 3 ⋅ 10^−3^ *μm*^3^, with approximately 400.000 nodes. Both the cell and ECM are imported to ABAQUS, where the FE analysis is performed, although any other FE solver would be suitable.

#### Definition of materials

The presented methodology allows the definition of any type of material for any domain. As a first approach, we assume that both the cell and ECM behave as hyperelastic Neo-Hookean isotropic materials [21, 43, 44]. In this case, the strain energy density function *Ψ* is defined by:

$$\begin{array}{c} \Psi =\Psi_{vol}+\Psi_{iso}=C_{10}\left(\bar{I_{1}}-3\right)+\frac{1}{D_{1}}{\left(J-1\right)}^{2} \end{array}$$ (3)

where Ψ_*vol*_ and Ψ_*iso*_ are the volumetric and isochoric components of the strain energy density function, respectively. *C*_10_ and *D*_1_ are the material constants, $\bar{I_{1}}$ is the first invariant of the deviatoric strain tensor and *J* is the elastic volume ratio. Thus, to define Neo-Hookean materials, both the *C*_10_ and *D*_1_ parameters must be known, and they can be formulated as functions of the initial shear and bulk modulus. In this work, we define *C*_10_ equal to 20 kPa and *D*_1_ equal to 0.005 kPa^−1^ for the cell and the ECM [45]. Both the collagen gel and cell are assumed to be almost incompressible materials [36]. As a first approach, we assume that the cell adapts its cytoskeleton to have the same mechanical properties as those of the ECM [46].

#### Recovery of cell surface tractions

The information provided by the 3D displacement field (**u**_*real*_) is used to recover the cellular traction forces by applying the computational loop explained before. The algorithm is implemented in Python, and the FEM analysis is performed via the ABAQUS software (Dassault Systemes, Vlizy-Villacoublay, France). The remeshing software used is Tetgen [42].

As the problem is solved over different steps, it is necessary to gather the forces in the final configuration once convergence has been achieved. The postprocessing code allows for computing and visualizing both the surface tractions and the error in Paraview [47]. Since this methodology is iterative, the total surface tractions exerted by the cell are computed from the mathematical manipulation of those obtained during each iteration. Since the analysis is implemented for large deformations, the logarithmic strains are postprocessed to obtain the final surface traction distribution. During each iteration, the geometry is discretised in a different mesh (different configuration), so it is necessary to interpolate and add the logarithmic strains between meshes to merge these results for the final mesh. Thus, the results are interpolated from the integration points to each node in the cell surface. Finally, the stress field is obtained by deriving the strain energy function of the Neo-Hookean material formulation Eq (3).

Therefore, the stress is formulated as:

$$\begin{array}{c} \sigma =\;\sigma_{vol}+\sigma_{iso}=2\frac{\partial \Psi_{vol}\left(J\right)}{\partial C}+2\frac{\partial \Psi_{iso}\left(C\right)}{\partial C}=\frac{2C_{10}}{J}\left(\bar{b}-\frac{I_{1}}{3}I\right)+\frac{2}{D_{1}}\left(J-1\right)I\; \end{array}$$ (4)

where *σ*_*vol*_ and *σ*_*iso*_ are the volumetric and isochoric stress components, respectively. **C** is the right Cauchy-Green tensor, and **I** is the identity tensor.

To compute the stress, we must know the logarithmic strains in the final configuration for each element of the mesh. The required variables are computed from the logarithmic strains in the current configuration.

The logarithmic strains are defined by ln **V**, where $V=\sqrt{F\cdot F^{T}}$ is the left stretch tensor. In this way, we can obtain the left Cauchy-Green tensor **b**, which is defined by **b** = **F** ⋅ **F**^**T**^. We call $\bar{b}=J^{-\frac{2}{3}}\cdot b$ the modified left Cauchy-Green tensor, where $J=\sqrt{det(b)}$. The first invariant is defined by $\bar{I_{1}}=tr\left(\bar{b}\right)$. Finally, the surface traction field is computed as **t** = *σ* ⋅ **n**.

### Analyses performed

The aim of the proposed methodology is to recover the surface traction field exerted by a cell that yields a known displacement field. To test the methodology, a benchmark problem is created first (see S1 File). Second, a theoretical direct analysis was conceived so that the results obtained could be compared with the direct results. To validate the method, it is necessary to develop a computational case in which the solution is known to properly validate the methodology and to isolate the validation of the methodology from biological and *in vitro* uncertainties. To this end, an *in silico* case is created in which a spherical cell is assumed to be the undeformed configuration. For this analysis, the cell exerts traction forces to undergo finite deformation (up to 50%). The forces are defined to cause finite deformations over the surface of the sphere. The displacements obtained are compared with the computed displacements, and thus, the error can be quantitatively measured.

After validation is performed, a real cell geometry with real (experimentally measured) displacements is tested. The displacement field **u**_*real*_ is estimated via confocal microscopy and bead tracking. The studied cell geometry has a size of approximately 60 *μm* with three long protrusions, and it is embedded in the centre of a 102 x 102 x 30 *μm* mesh domain, with refinement performed on the cell surface to capture the tractions on the spindle-like protrusions with certainty. We define the threshold *δ* to be 15%, and this threshold should be achieved in 90% of the nodes.

## Results

### Computed traction field problem

**Fig 3:**
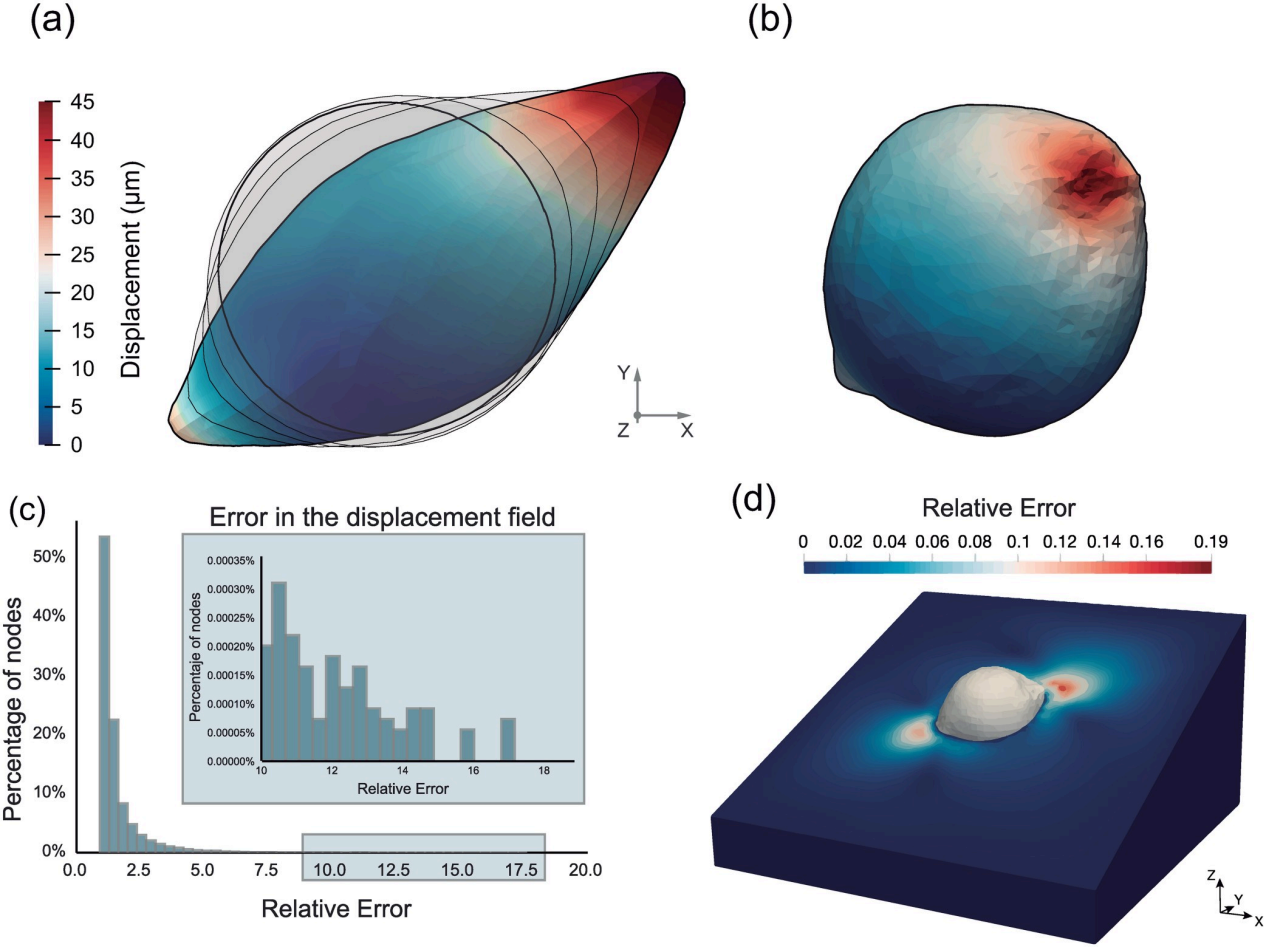
Displacement magnitude (*μ*m) at the cell surface. (a) Displacement field **u**_*real*_ in the undeformed configuration of the direct problem. The different configurations obtained by solving the direct problem are shown in the background in gray. (b) Displacement field **u**_*computed*_ in the deformed configuration of the inverse problem. The inverse problem aims to achieve the same configuration as the direct problem’s undeformed configuration. The displacement field obtained via the proposed methodology is remarkably similar to the displacements suffered by the sphere in the direct problem. (c) Error distribution obtained by using the inverse method. More than 70% of the nodes present relative errors lower than 1%. The maximum error obtained is 19.05%. (d) Relative error plotted all over the ECM.

First, to validate our methodology, we use the FEM to solve a direct problem with a set of known surface tractions **T**_*real*_. In order to simplify the validation process, the cell geometry is assumed to be a sphere with a diameter of 80 *μm*. Cell forces on the order of tens of nanonewtons are imposed at pseudo-randomly chosen discrete locations on the body surface of the sphere (randomly distributed inside the chosen areas). The sphere is embedded in a rectangular prism, simulating an ECM in which external surfaces are constrained (see S1 Fig). As the surfaces are distant enough from the cell, this condition does not affect the recovery of the cell traction forces. In this theoretical experiment, the geometry is meshed with tetrahedral elements with sizes of 17.5 *μm*^3^ to verify the accuracy of the method with this element type. Fig 3 shows the deformed and undeformed configurations of the direct problem. The displacement field is made particularly large (compared to the sphere radius) to test the methodology with respect to finite material deformations.

The results show similar geometries when comparing the obtained result with the undeformed geometry. Fig 3 depicts the displacement field suffered by the sphere surface. Specifically, the largest displacement computed is 47 *μm*, which is higher than the sphere radius (40 *μm*).

The maximum displacement obtained in the direct case is located mainly on one side (see Fig 3a). For this particular case, the analysis reaches convergence after five iterations with four intermediate meshes that were automatically generated.

**Fig 4:**
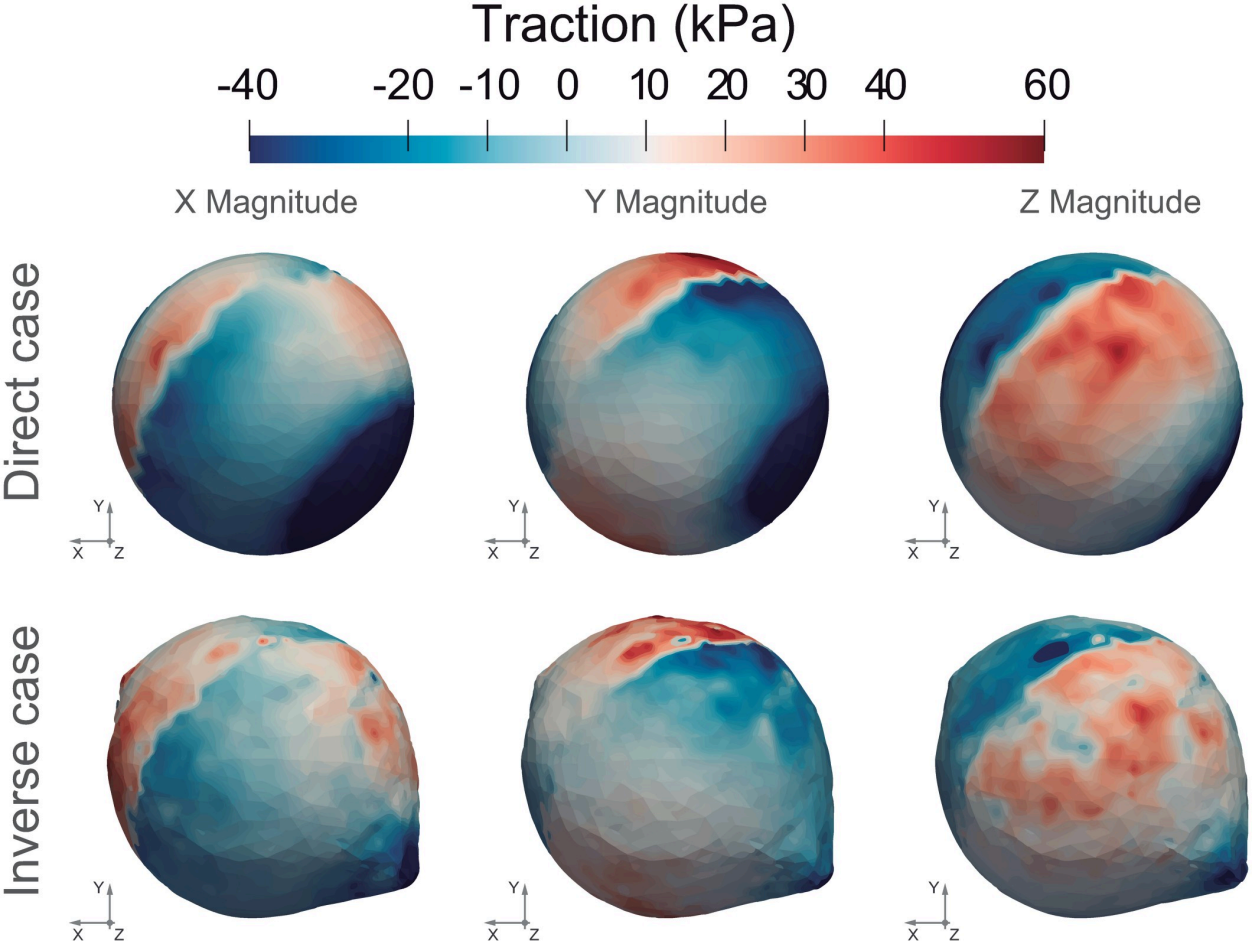
Surface tractions (kPa). Comparison between the direct computed surface traction fields (kPa) (top) and the recovered one via the numerical resolution of the inverse problem (kPa) (bottom). The undeformed configuration is shown in the direct case. The traction forces obtained through the inverse analysis are depicted in the deformed configuration, since this one represents the initial geometry of the direct case.

The spherical shape is reasonably recovered, with some bumps in zones of maximum deformation (Fig 3). To quantitatively compare the results obtained for the inverse problem with those obtained for the direct problem, it is essential to obtain the surface tractions in the direct problem. The simulated surface tractions (direct problem) and those obtained by solving the inverse problem for the case described above are shown in Fig 4.

The maximum of the computed surface tractions in each direction is approximately 70 kPa, which is in agreement with the traction field imposed in the direct problem. The surface tractions are similarly distributed and have the same magnitudes as those of the direct problem.

Additionally, since the number of nodes is very high and the distribution of displacements is highly asymmetric, the nodal displacements are compared. The relative nodal error distribution can be observed in Fig 3. The highest error obtained is 19.05%; however, the majority of the nodes present very slight deviations. In fact, ninety-five percent of nodes have errors lower than 2.62%.

### *In vitro* cell geometry and displacements field

After testing the methodology in a theoretical problem, the presented approach is applied to a real scenario. Thus, a real cell geometry with a displacement field obtained from the TFM experiments is analysed.

**Fig 5:**
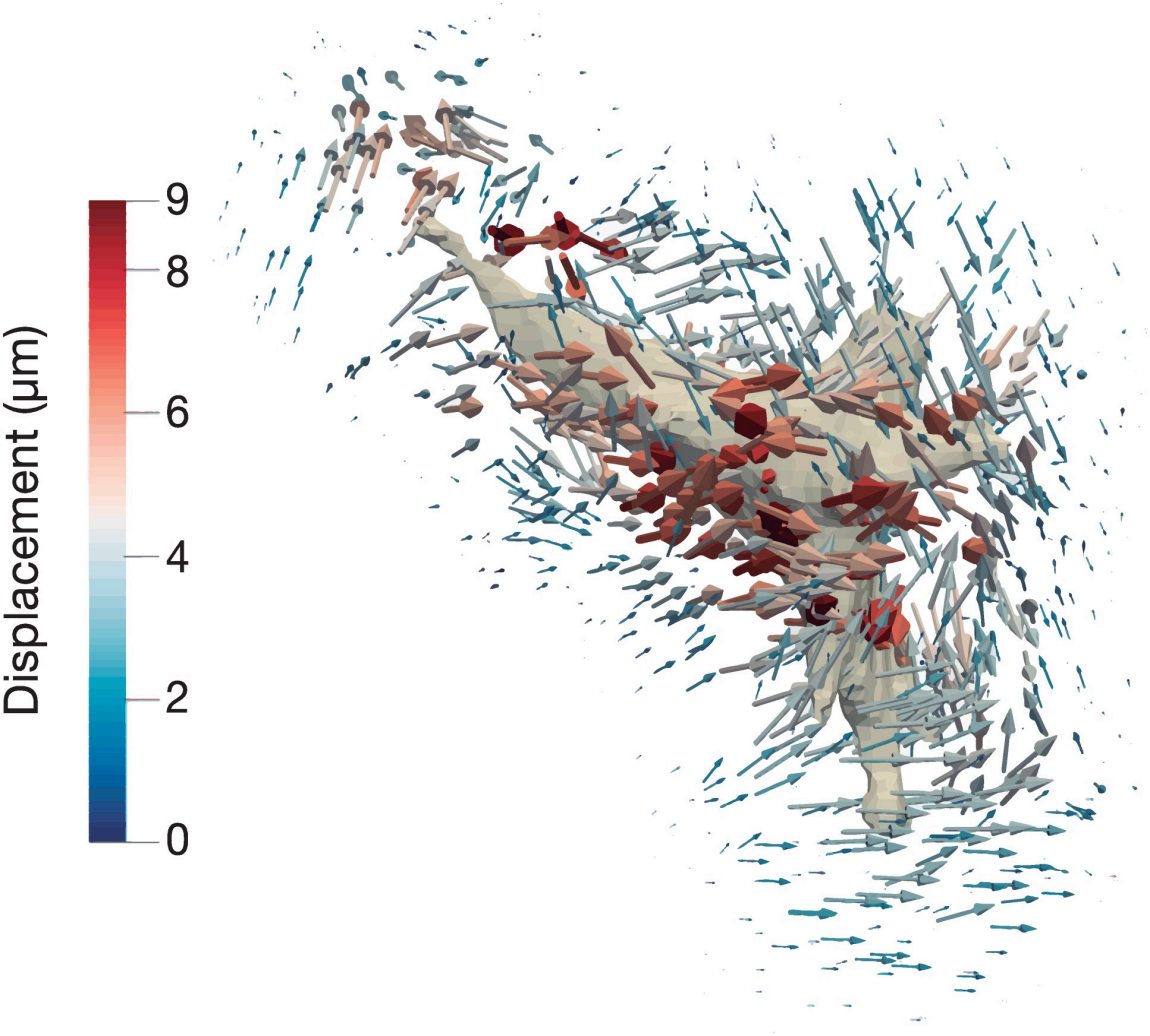
Displacement field generated by the cell in the *in vitro* TFM experiment. Bead displacement trajectories of a normal human dermal fibroblast embedded in a collagen matrix obtained in the lab experiments, color coded by magnitude.

The ECM displacements are mainly concentrated around the cell, diminishing gradually as the distance increases (Fig 5). Nevertheless, the whole ECM is simulated to avoid numerical discontinuities, as the gel contour is constrained as a rigid solid to restrict movement.

The geometry is first discretized in a voxel mesh, derived from the segmentation of the image stacks.

**Fig 6:**
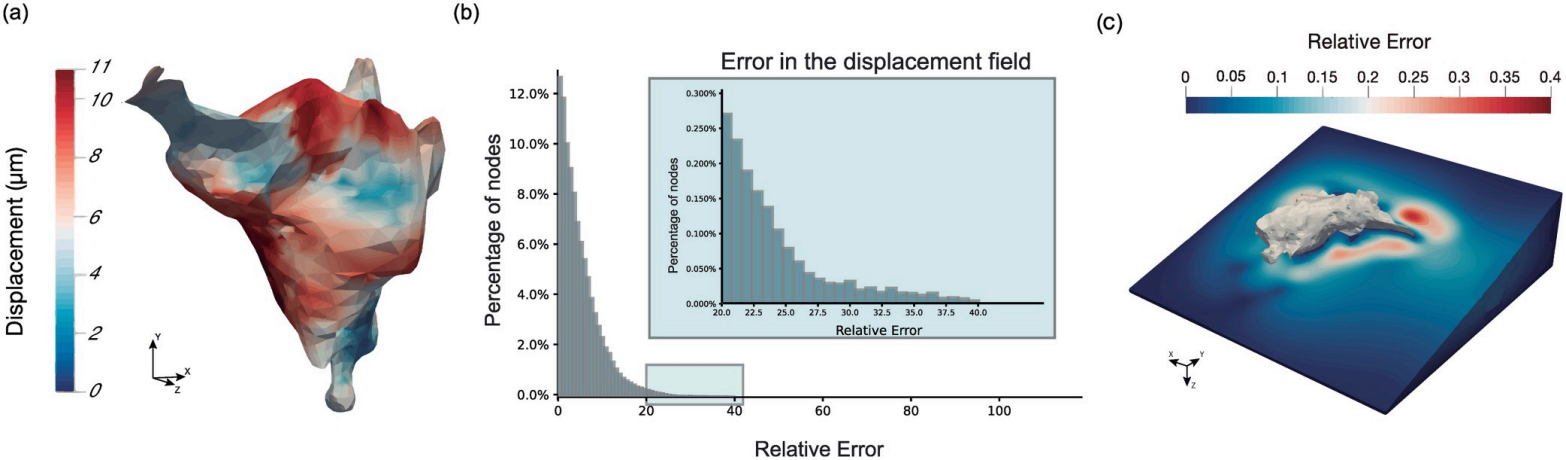
Measurements of displacements obtained. (a) Displacement (*μm*) field obtained at the cell surface. (b) Displacement error distribution. The maximum relative error obtained is 39.95%. The error obtained at the 90th percentile is 11.28%. Maximum errors (higher than 35%) appear in less than 0.05% of the nodes. (c) Relative error plotted all over the ECM.

After the experimental analysis, both the displacement field and the deformed configuration are used as inputs for the inverse problem solver. The displacements obtained at the cell surface are depicted in Fig 6. The maximum displacements are located at the center of the cell, where we hypothesise that the nucleus is located. Two out of the three protrusions are moving; nevertheless, the third protrusion is not relaxing at all.

**Fig 7:**
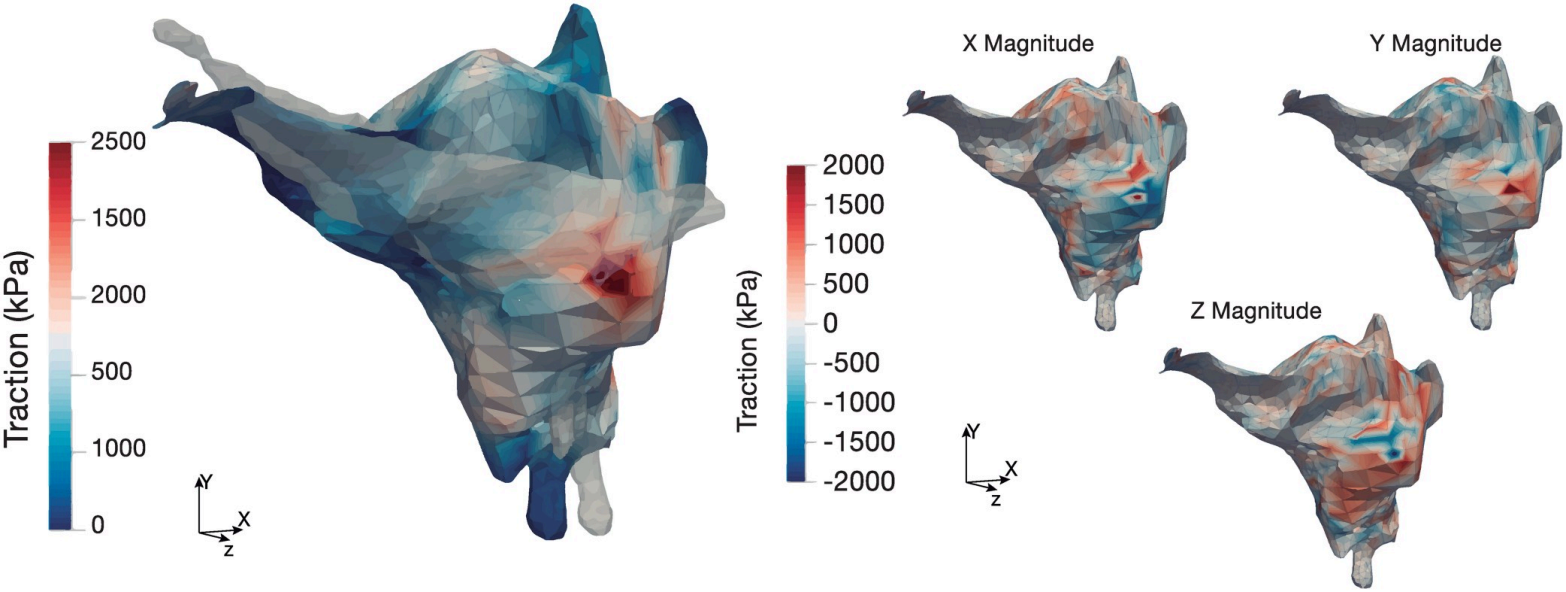
Cell surface traction components (kPa) computed at the cell surface. On the left, cell traction magnitude at the deformed cell surface. Overlaid in white, the undeformed cell configuration. On the right, the three different components of the cell traction forces.

The surface tractions obtained using the proposed methodology are depicted in Fig 7. The maximum traction force measured is 2500 kPa.

A hypothesis regarding the model is that the whole surface of the cell can exert forces, since the locations of the adhesion points are unknown. For this reason, since the displacements of the real case are uniform throughout the gel, the traction forces obtained are distributed over the cell surface, although they are most concentrated where the displacements are highest.

For this particular example, the maximum displacement obtained is equal to 12.24 *μm*, and the analysis converges in the 54th iteration. Fig 6 represents the distribution of the relative nodal displacement errors. This distribution is highly asymmetric, with 90% of the nodes having errors below 12% according to the criterion established in Eq 2. Relevant errors occur in less than 0.05% of the nodes.

## Discussion and conclusions

In this work, we develop an automatic methodology to compute the surface tractions exerted by a cell embedded in a 3D environment. To test the proposed methodology, we analyse two cases. The first one is a theoretical case used to validate the model, which recovers the computed traction forces within a relatively small number of iterations, generating a maximum error of 19.05% in terms of displacement and errors lower than 2.62% in ninety-five percent of the total nodes. The second case, in which a real cell is embedded in the ECM, leads to a maximum relative error of 39.95%. These results show the efficiency of the proposed methodology. It is worth noting that these percentages totally depend on the criterion established to calculate the nodal errors (Eq 2), which are now normalised against the maximum displacement. Other criteria, such as the average or a specific percentile of the displacement magnitudes, could have been used. In any case, the distribution of errors would remain the same, with few nodes having relevant deviations.

The first case studied was designed to test the methodology in a very unfavourable scenario. That is, the simulated traction forces are large enough to increase the deformations up to 110%. However, the computed surface tractions obtained in the direct and inverse simulations are comparable, both in magnitude and in terms of the application points.

Compared to the existing methods in the literature, our model presents some strengths worth highlighting. First, our methodology is fully automatised, providing a useful tool to complement laboratory experiments. The input data for the algorithm are the images (z-stacks) taken during the TFM experiment at two different time points, and the algorithm produces the cell surface tractions as output. It is worth noting, however, that the computation time is in the range of several hours. In any case, the methodology is applicable to any cellular type or to any entity exerting forces, since it is totally independent of the initial geometry, material or displacement values.

The use of real cell geometries introduces difficulties with regard to the resolution of the problem since automatic segmentation does not generally produce smooth 3D surfaces; this is primarily due to the discontinuities between z-slices. Nevertheless, the method proposed here is able to overcome these discontinuities through an automatic remeshing algorithm that updates the elements to ensure optimal quality. In fact, element size is defined by the resolution of the images captured during the TFM experiment, thus, one element of the mesh corresponds to one voxel from the image data. The error obtained is not dependent on the initial mesh element size but controlled by the threshold selected. A more refined mesh might help to increase the accuracy of the recovered traction field at the expense of increasing the computational cost of each iteration. Additionally, if a more refined mesh than the image resolution is used, an interpolation process of the displacements would be necessary, and this could be a source of non-measurable errors.

Since we do not have any information regarding the focal adhesion sites (FASs), we assume that the entire cell surface is capable of exerting traction forces. Some authors assume the locations of focal adhesions to simplify the problem [38]. With the method proposed here, no simplification is made, and the force distribution that minimises the overall error is obtained. However, if this information were available, the locations of the FASs could be easily incorporated into the model, refining the results and accelerating the computation process.

One of the most important benefits of this method is that the inverse problem is solved for large deformations. Soft substrates employed in TFM can undergo deformations beyond the linear elastic regime, thus requiring a finite-strain TFM approach [28]. [44] emphasised the need for the latter approach, as the error incurred by the traction stresses due to linearisation exceeds 30% for moderate cell-induced substrate deformations (strains on the order of 50%). However, the implementation of this hypothesis is not trivial since inverse problems present numerical complexities, especially when they involve large deformations. Therefore, other methodologies are limited to linear strains [33, 34], while our approach allows for the computation of cell forces for a regimen of finite strains.

Materials with nonlinear elastic behaviours have been modelled, although there is no restriction on the use of any other constitutive model assumptions. Most existing models [33, 48] presented in the literature use linear elastic material properties for the cell and gel domains. However, to simulate fibrillar collagen matrices, it is essential to use a material with nonlinear behaviour. The parameters are chosen in accordance with [45]; nevertheless, they can be modified. Increasing the C10 parameter results in an increase in the traction forces since we are increasing the stiffness of the cell and ECM. A decrease in this parameter reduces the traction forces. Palacio *et. al*. [34] remarked on the importance of considering the nonlinear properties of the ECM, showing that large errors in the recovered traction field come from the linearity assumption. Additionally, Dong *et. al*. [35] concluded that the error in the recovered traction field is sensitive to whether the nonlinear effects in the TFM problem are neglected. Recently, some authors have modelled the fibrillated behaviour of the ECM. Song *et. al*. [26] proposed a fiber-based constitutive 3D model using real cell geometries and synthetic traction fields, relying on the hypothesis that cellular tractions can induce a strong fiber realignment. In the present work, the choice of the Neo-Hookean material formulation is made as a first approximation and might represent some limitations with regard to modelling the behaviour of the collagen-based matrix. The tensile behaviours of collagen fibres are properly modelled with a nonlinear material approximation, such as the Neo-Hookean material formulation implemented. The compression behaviour is not accurately modelled by this material formulation. Therefore, the implementation of a more suitable material model, such as the Steinwachs model [36], is proposed as a future line of research. Regardless, from our point of view, this change would not have a strong influence on the results obtained here. The present work applies the same material behaviour for both the cell and the ECM, since previous works [38] stated that this simplification for the range of parameters studied has only a minor effect on the resulting traction values. In fact [46], stated that the stiffness of the cytoskeleton changes depending on the ECM in which it is embedded.

Since the displacement caused by the cell is large, the change in the current configuration must be accounted for, and this introduces the so-called geometric nonlinearity to the problem. To this end, we split the whole problem into smaller parts, and therefore, the geometry must be uploaded and remeshed during each iteration. At the end of the simulation, we need to interpolate the results obtained from one mesh to the next mesh over the entire analysis. To compute the traction forces in the deformed configuration, all the previous configurations must be considered. The final tractions are not equal to the sum of the tractions computed during each iteration. We must collect the logarithmic strains and sum them over different configurations.

Another advantage of the proposed method is that it can be easily implemented in any type of FE software and can be extended to any type of contractile cell. Moreover, the problem of recovering tractions is not limited to applications in biomechanics; this has also been studied in structural dynamics [41].

To reconstruct the 3D geometries of NHDF cells embedded within a collagen gel, confocal microscopy imaging with laser scanning is used. Subsequently, the obtained z-slices are segmented to generate single 3-D models of both the cell and gel domains. These real geometries are then meshed and used to generate the FE model that is then used for the numerical simulations. However, the imaging technique still presents some limitations, such as low temporal sampling and the risk of photobleaching/phototoxicity. To avoid artifacts, only displacements of the cell action area are tracked. This action area is assumed to be 10 *μm*. Although there are important works that state that forces are transmitted beyond this distance [49], testing shows that most of the displacements out of this zone are negligible except for sparse peaks that are assumed to be artifacts. Including the whole raw dataset does not noticeably affect the results but drastically increases the instability.

Computationally, there are some relevant works that have extracted cell traction forces from the displacement of the ECM during the TFM experiment. Nevertheless, most of these works [37, 44, 50, 51] developed a semi-3D approach, where the cell is placed at the top of the gel and is not embedded in the ECM.

The relevance and novelty of the presented methodology lies in the fact that none of the previously presented works incorporated all of the following features at once: 3D-TFM, large deformations, real experimental data and automatisation. Some previous works calculated the cell traction forces but assumed the hypothesis of small strains [33, 38, 48]. Furthermore, those who worked under the finite strain regime did not extract the traction force field but rather just the strain tensor [12]. Only a few attempts have been made to recover cellular forces by considering large deformations [35]. However, the authors considered a computationally made displacement field, not an experimental field based on real cells. Toyjanova *et. al*. [44] presented a methodology that recovers the displacement field directly from TFM experiments and uses it as an input for the inverse problem solver. However, the cell is not completely embedded in the ECM but leaned on a gel.

In summary, we have presented a novel methodology to automatically recover cell surface tractions from the displacement field, even under the finite strain hypothesis and using nonlinear materials. We have validated its potential against a theoretical case and applied it to a real cell experiment scenario with experimentally measured displacements. Our method is flexible enough to be applicable to any kind of geometry and material property, and it has shown robustness even under extreme strain conditions, thus overcoming the limitations usually found in the literature with respect to similar procedures.

## Supporting information

S1 File A benchmark problem for inverse analysis in TFM. (PDF)

S1 Fig Boundary conditions for a sphere embedded inside the ECM (computed traction field problem). The sphere is embedded inside the ECM. A set of known traction forces is applied on cell surface.
(TIF)

S2 Fig Displacement magnitude (μm) at the cell surface. (a) Displacement field **u**_*real*_ in the undeformed configuration of the direct problem. The different configurations obtained solving the direct problem are shown in the background in grey. (b) Displacement field **u**_*computed*_ in the deformed configuration of the inverse problem. The inverse problem aims to achieve the same configuration as the direct problem’s undeformed one. The displacement field obtained via the proposed methodology is quite remarkably similar to the displacements suffered by the sphere in the direct problem. (c) Error distribution obtained by using the inverse method. More than 70% of the nodes present an absolute error lower than 1%. The maximum error obtained is 19.05%. (d) Relative error plotted all over the ECM.
(TIF)
