# Correction: A new 3D finite element-based approach for computing cell surface tractions assuming nonlinear conditions

**DOI:** 10.1371/journal.pone.0316471

**Published:** 2024-12-20

**Authors:** Silvia Hervas-Raluy, Maria Jose Gomez-Benito, Carlos Borau, Mar Cóndor, Jose Manuel Garcia-Aznar

The name of the third author is incorrect. The correct name is Carlos Borau. The correct citation is: Hervas-Raluy S, Gomez-Benito MJ, Borau C, Cóndor M, Garcia-Aznar JM (2021) A new 3D finite element-based approach for computing cell surface tractions assuming nonlinear conditions. PLOS ONE 16(4): e0249018. https://doi.org/10.1371/journal.pone.0249018.
