# Supplementary material for: A new 3D finite element-based approach for computing cell surface tractions assuming nonlinear conditions
Source: PLoS One. 2021 Apr 14;16(4):e0249018. doi: 10.1371/journal.pone.0249018 (PMC8046236; doi:10.1371/journal.pone.0249018)
Supplement: S1 File — (PDF) [file pone.0249018.s001.pdf]

S1 File

A benchmark problem for inverse analysis in TFM

To further test the methodology proposed, a benchmark problem is numerically discussed. We implement a uniaxial traction test, where some forces are applied in the central nodes simulating the forces exerted by one cell in the longitudinal direction. The model consists of one 200x10x10 μm bar discretised into 21 voxels (see Fig. S3). The central voxel represents one cell (element in dark green), while the remaining 20 voxels, 10 to each side, simulate the ECM. We assume that both cell and ECM behave as hyperelastic Neo-Hookean isotropic materials. In this analysis, we set the value of the parameters C_{10} and D_1 to 23.077 kPa and 0.02 kPa^{-1} respectively for the cell and 2.3077 kPa and 0.2 kPa^{-1} for the ECM. A force of 5000 nN is imposed to each of the eight nodes of the cell voxel as shown in Figure S3. Boundary conditions were fixed in order to simulate a uniaxial problem in which the cross section area is not allowed to change its shape. Thus, the displacements only occur in the longitudinal direction. The maximum displacement obtained in the direct analysis was 8.115μm, which occurs in the cell.

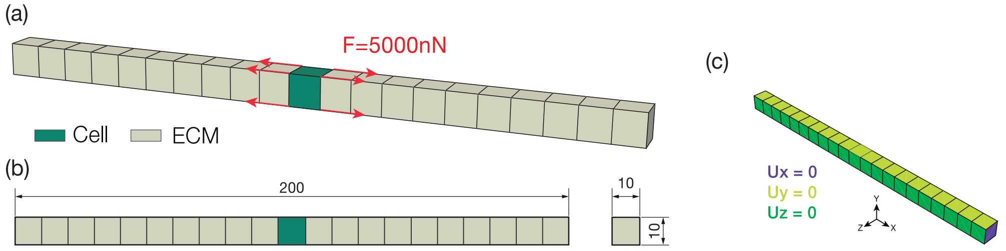

Fig S3. Uniaxial traction benchmark analysis. (a) Geometry of the model. In dark green colour, the voxel representing a cell, in light green those voxels simulating the ECM. Forces due to cell activity are imposed to each of the cell nodes (red arrows). (b) Dimensions of the model in microns. (c) Boundary conditions applied during the analysis. Both y and z displacements are forced to be zero. At the ends of the bar, the x-displacement is prevented to avoid the movement as a rigid solid.

The resulting displacement field in the ECM obtained from the forward problem, was used as input for the inverse method analysis. After running six iterations, the analysis reached convergence, obtaining a maximum displacement of 8.11467μm in the cell. The maximum relative error measured in displacements was 0.022%. Figure S4 summarizes the results obtained in this benchmark problem.

In the TFM analysis, the aim is to obtain the traction forces to relate them with the forces exerted by cells. However, it is important to distinguish among the different variables related to the mechanical effort exerted or sensed by cells and ECM that can be numerically computed: the forces exerted by the cell, surface tractions (force per area), the stress sensed by the cell and the strains of both cell and ECM. Therefore, it is essential to define the specific output or variable that we aim to recover from this inverse analysis. The straightforward result is obtaining the strains experienced by the ECM. For example, Peñas *et. al.* [1] recovered the Green-Lagrange strain tensor in the

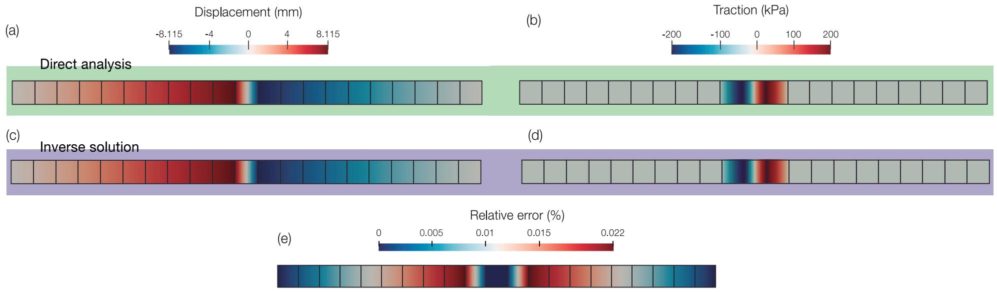

Fig S4. Uniaxial traction benchmark analysis. (a) Displacement obtained in the direct analysis. (b) Tractions imposed in the direct analysis. (c) Displacement recovered after running the inverse method. (d) Tractions recovered in the inverse analysis. (e) Relative error obtained measured in displacements. Maximum errors are located close to the cell and their value is 0.022%.

ECM. Using the strain tensor and the energy deformation function, the stress tensor can be also computed in the ECM. Firstly, using the density energy function of the ECM, the stress exerted by the ECM into the cell is calculated. Similarly, if the cell mechanical properties are known, using the density energy function of the cell, the stress exerted by the cell into the ECM is obtained. Thus, only the second one is taken into account since the aim of the TFM inverse methodology is to obtain the forces exerted by the cell. After computing the stress tensor, another output available is the surface traction forces. These output is obtained in the majority of the inverse methods presented in the literature [2–4]. To obtain surface tractions it is necessary to calculate the normal vectors to the planar surface. Concentrated forces can be calculated, but in this case the area of each element has to be obtained. Following the assumption that two different stress tensors can be computed ($\sigma_{ECM \to cell}$, the stress the ECM exerts on the cell and $\sigma_{cell \to ECM}$, the stress the cell exerts on the ECM), also two different forces can be calculated. The sum of both forces represents the total forces exerted by the cell.

Following this benchmark problem, all these outputs can be easily checked. Since the problem is uniaxial, only the longitudinal direction results are shown. After performing the inverse analysis, the stress obtained is distributed between the cell and the ECM: $\sigma_{ECM \to cell} = -1.438$ kPa and $\sigma_{cell \to ECM} = 198.561$ kPa. Therefore, the force applied by the cell in the interface between the cell and the ECM, since there is no change in the cross section area due to boundary conditions is equal to 19999.96 nN, i.e. 4999.99 nN per node.

References

1. Jorge-Peñas A, Bové H, Sanen K, Vaeyens MM, Steuwe C, Roeffaers M, et al. 3D full-field quantification of cell-induced large deformations in fibrillar biomaterials by combining non-rigid image registration with label-free second harmonic generation. Biomaterials. 2017;136:86–97. doi:10.1016/J.BIOMATERIALS.2017.05.015.

2. Legant WR, Miller JS, Blakely BL, Cohen DM, Genin GM, Chen CS. Measurement of mechanical tractions exerted by cells in three- dimensional matrices. Nature Methods. 2010;7(12):969–971. doi:10.1038/nmeth.1531.

3. Holenstein CN, Lendi CR, Wili N, Snedeker JG. Simulation and evaluation of 3D traction force microscopy. Computer Methods in Biomechanics and Biomedical Engineering. 2019;22(8):853–860. doi:10.1080/10255842.2019.1599866.

4. Dong L, Oberai AA. Recovery of cellular traction in three-dimensional nonlinear hyperelastic matrices. Computer Methods in Applied Mechanics and Engineering. 2017;314:296–313. doi:10.1016/j.cma.2016.05.020.
