# Supplementary figures and images for: A new 3D finite element-based approach for computing cell surface tractions assuming nonlinear conditions
Source: PLoS One. 2021 Apr 14;16(4):e0249018. doi: 10.1371/journal.pone.0249018 (PMC8046236; doi:10.1371/journal.pone.0249018)

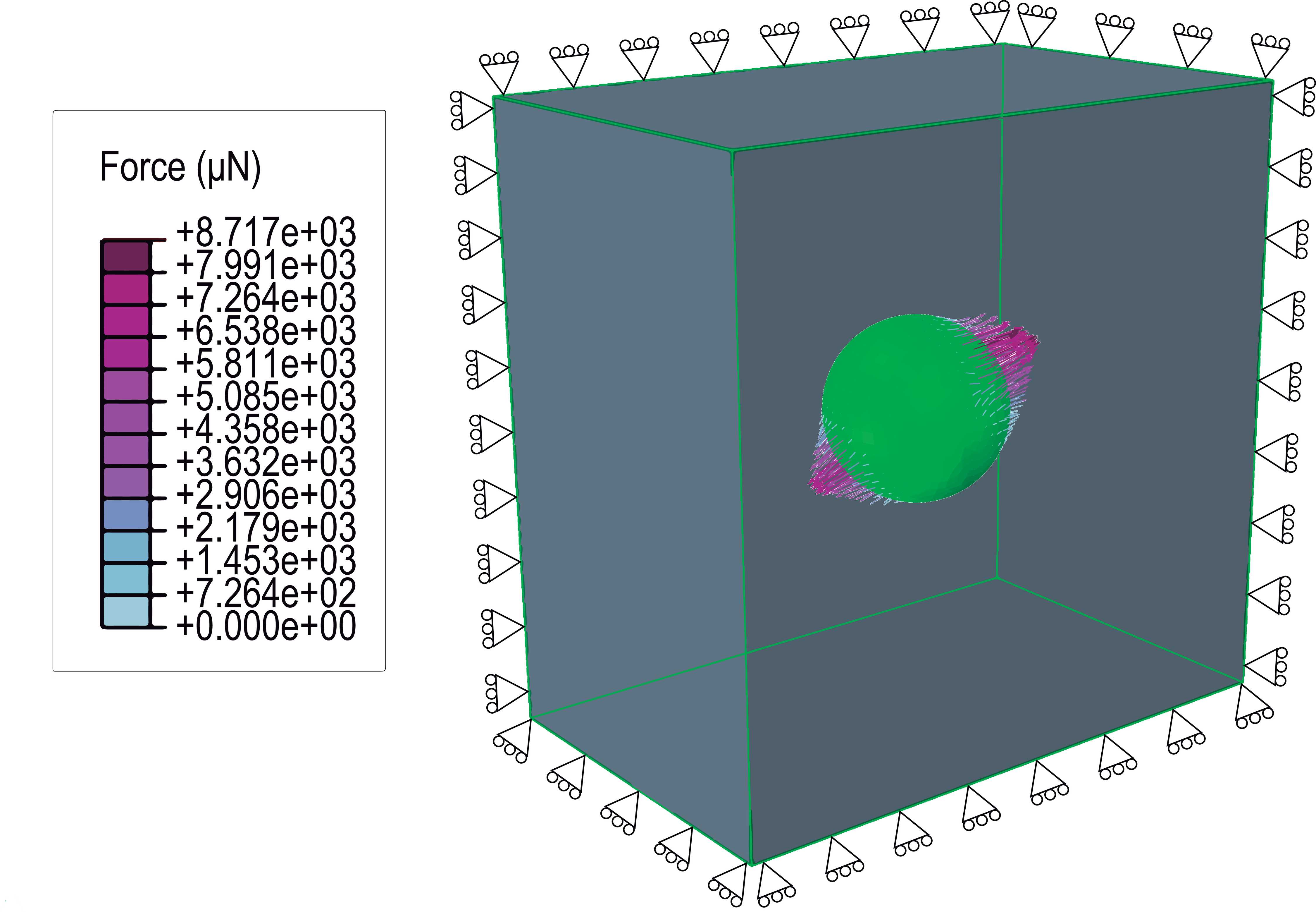

Supplement: S1 Fig — The sphere is embedded inside the ECM. A set of known traction forces is applied on cell surface. (TIF) [file pone.0249018.s002.tif]

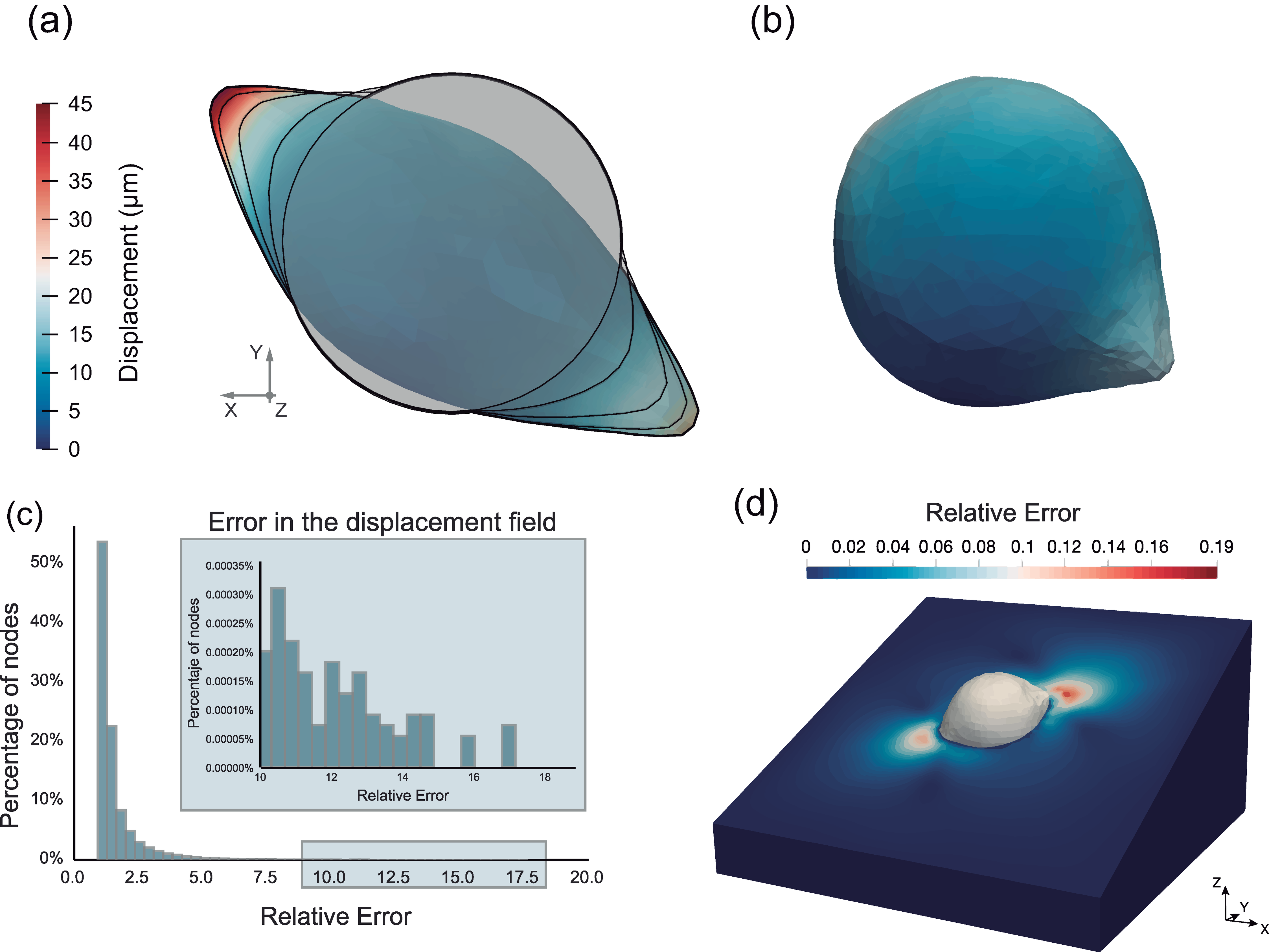

Supplement: S2 Fig — (a) Displacement field ureal in the undeformed configuration of the direct problem. The different configurations obtained solving the direct problem are shown in the background in grey. (b) Displacement field ucomputed in the deformed configuration of the inverse problem. The inverse problem aims to achieve the same configuration as the direct problem’s undeformed one. The displacement field obtained via the proposed methodology is quite remarkably similar to the displacements suffered by the sphere in the direct problem. (c) Error distribution obtained by using the inverse method. More than 70% of the nodes present an absolute error lower than 1%. The maximum error obtained is 19.05%. (d) Relative error plotted all over the ECM. (TIF) [file pone.0249018.s003.tif]
